# Direct and indirect relationships between structural empowerment, professional competence, thriving at work and perceived stress symptoms: a cross-sectional correlational study on hospital nurses in a Chinese province

**DOI:** 10.1136/bmjopen-2025-100696

**Published:** 2025-12-25

**Authors:** Lijuan Xu, Annika Nilsson, Kewen Zhu, Maria Engström

**Affiliations:** 1 Medicine college, Lishui University, Lishui, Zhejiang, China; 2 Department of Caring Science, Faculty of Health and Occupational Studies, University of Gävle, Gävle, Gavleborg, Sweden

**Keywords:** Nurses, Caregivers, Cross-Sectional Studies, Health Services, Stress, Psychological, Clinical Competence

## Abstract

**Abstract:**

**Objectives:**

Work stress is a threat to the well-being of nurses in China and also globally, and many studies have been conducted on the risk factors for stress symptoms. However, research on the process and mechanism between work environment risk factors and perceived stress symptoms among nurses remains limited. This study aimed to examine the direct and indirect relationships between nurse-rated structural empowerment, professional competence, thriving and nurses’ perceived stress symptoms.

**Design:**

This was a cross-sectional correlational study.

**Setting and participants:**

This study was conducted between April and October 2023, and 2172 nurses at three general hospitals in a Chinese province were recruited.

**Method:**

A questionnaire that included demographic information, the Conditions of Work Effectiveness Questionnaire, the Nurse Professional Competence Scale, the Thriving at Work Scale and one factor from the Psychosomatic Health Aspects Questionnaire that measured perceived stress symptoms was used. The PROCESS macro (model 4) with a parallel mediation model and bootstrapping tests was conducted to examine the direct and indirect relationships of structural empowerment, professional competence and thriving at work on perceived stress symptoms.

**Result:**

The regression analysis showed that structural empowerment, professional competence and thriving at work were negatively related to stress symptoms (β=−0.42, p<0.001; β=−0.06, p=0.009; β=−0.28, p<0.001, respectively), after controlling for marital status (yes/no), clinical experience and night shift (three groups). The results of the bootstrap analysis with a parallel mediator model revealed indirect relationships between structural empowerment and stress symptoms through both professional competence and thriving at work (β=−0.033 (95% CIs −0.059 to –0.009); β=−0.164 (95% CIs −0.204 to –0.126), respectively).

**Conclusions:**

Structural empowerment was related to stress symptoms both directly and indirectly via professional competence and thriving at work. The findings suggest that managers should alleviate nurses’ stress symptoms through measures aimed at providing access to empowering structures, strengthening professional competence and promoting thriving at work.

**STRENGTHS AND LIMITATIONS OF THIS STUDY**

- A parallel mediation analysis was performed to identify the relationships among structural empowerment, professional competence, thriving at work and stress symptoms.
- The links with existing theory and models support our findings.
- A cross-sectional design was used which makes it challenging to draw conclusions about cause-and-effect relationships.
- Participants were drawn from 121 departments within three hospitals located in one region of China, which may have led to potential clustering effects and restricted the generalisability of the results.
- The mediation model explained a moderate share of variance in stress symptoms (R²=22.7%), indicating that additional unmeasured factors may account for the remaining variance.

## Introduction

 With the advances in medical care and the diversity of patients’ care needs, nurses face numerous hazards at work due to rapid changes in the healthcare system.^1^ As a substantial segment of the healthcare workforce, nurses experience high levels of work-related stress, which can adversely affect their well-being as well as patient safety.^2 3^ Regarding work stress and mental health, the structural empowerment theory has been suggested as an important strategy to promote employee well-being, such as reducing stress.^4 5^ In addition, the socially embedded model of thriving at work indicates that thriving is an important factor for positive mental health.^6^ Previous studies have also shown that structural empowerment^7^,^9^ and thriving at work^10 11^ are related to job stress among nurses. However, to the best of our knowledge, few studies have explored indirect relationships between structural empowerment and stress symptoms by investigating professional competence and thriving at work.

## Background

### Theoretical framework

In this study, the combination of Kanter’s Structural Empowerment theory and Spreitzer’s Socially Embedded Model of Thriving at work provided a comprehensive theoretical framework for examining the direct relationship between structural empowerment and perceived stress symptoms and the indirect relationships through professional competence and thriving at work.

### Kanter’s structural empowerment theory

Kanter’s structural empowerment theory defines empowerment as employees’ ability to access and mobilise necessary resources to achieve work goals.^5^ Structural empowerment arises from access to information, support, resources and opportunities, as well as from formal (eg, meaningful roles, decision latitude) and informal (eg, collegial networks) structures that facilitate these empowering conditions.^5^ In supportive organisations, both formal job structures (eg, meaningful roles, decision latitude) and informal structures (eg, collegial networks inside and outside the organisation) facilitate access to these empowering conditions, thereby promoting employee well-being and performance.^5^

### Relationships between structural empowerment, professional competence and psychological well-being (stress symptoms)

Many cross-sectional^89 11^,^14^ and some longitudinal^715^,^17^ studies have documented the link between staff access to structural empowerment and well-being. For example, Engström *et al*^11 12^ and Lundin *et al*^8^ found that structural empowerment was related to stress symptoms in cross-sectional studies, and Hagerman *et al*^17^ reported the same in a longitudinal study. Structural empowerment has also been linked to professional competence,^718^,^20^ although less research has been conducted on this relationship. Greater access to empowering work conditions has been found to enhance self-rated competence and improve work-related outcomes. Moreover, higher competence has been linked to improved well-being.^21 22^

Importantly, emerging evidence suggests that professional competence may function as a mediating mechanism through which work environment conditions influence psychological outcomes. For instance, Campos *et al*^22^ demonstrated that perceived competence mediated the effect of working conditions on burnout and stress among university professors, indicating that competence not only represents a personal resource but also a pathway linking structural factors to well-being. Specifically, research examining whether competence mediates the effect of structural empowerment on stress symptoms is sparse,^23^ and few studies to date have evaluated this pathway among clinical nursing staff. To address this gap, the present study proposes and tests a model integrating professional competence as a key mechanism linking structural empowerment to nurses’ psychological well-being.

Based on the theory of structural empowerment and earlier research, we formulated the following hypotheses:

Hypothesis 1: Higher structural empowerment is associated with fewer stress symptoms among registered nurses (RNs).

Hypothesis 2: Higher structural empowerment is related to higher competence among RNs.

Hypothesis 3: Higher professional competence is related to fewer stress symptoms among RNs.

**Figure 1:**
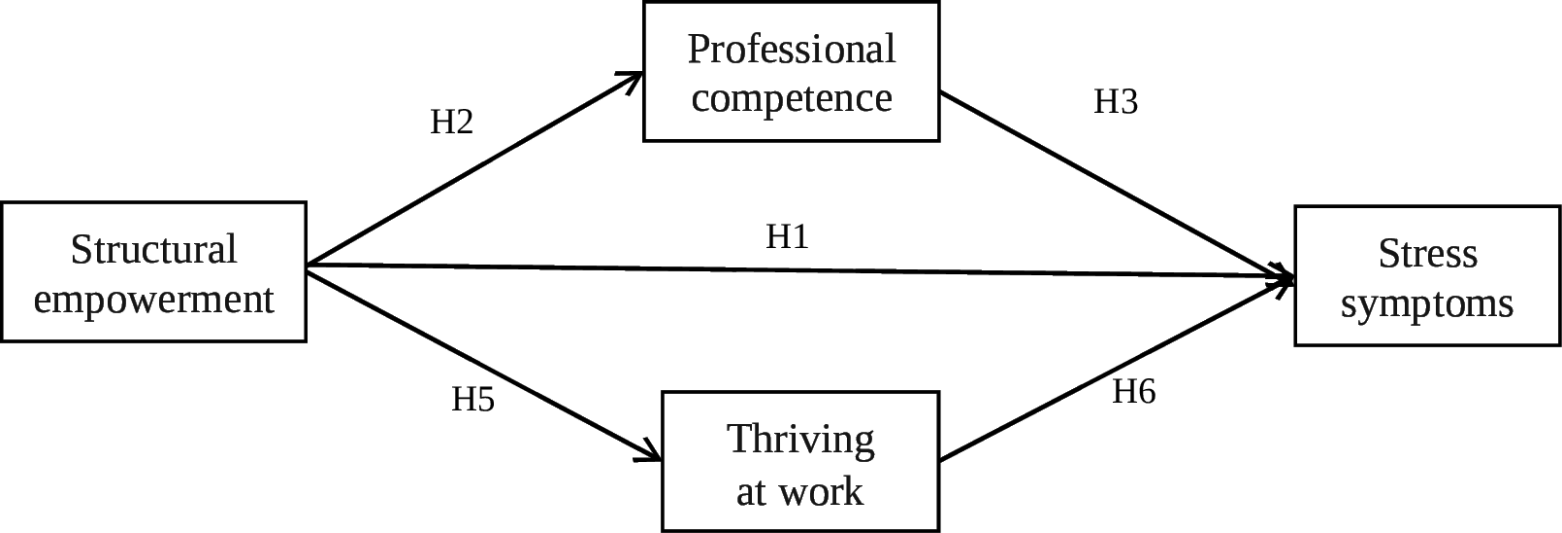
Hypothesised framework. H, hypothesis.

Hypothesis 4: There is an indirect relationship between structural empowerment and perceived stress symptoms through professional competence (figure 1).

### Spreitzer’s socially embedded model of thriving at work

Thriving at work refers to a psychological state of vitality (energy) and learning (growth in knowledge).^6^ According to Spreitzer’s model, thriving is fostered by work-generated resources—such as positive affect, meaning and relationships—and by supportive social structures that promote trust, information sharing and autonomy.^6^ These conditions contribute to cognitive growth and better health. Many of these antecedents overlap with Kanter’s concept of structural empowerment, which emphasises access to information, resources, support and opportunities.^5^ Guided by the Socially Embedded Model of Thriving, we propose that structural empowerment is related to nurses’ thriving at work, which in turn is related to their psychological well-being and reduces stress symptoms.

### Relationships between structural empowerment, thriving at work and psychological well-being (stress symptoms)

A meta-analysis on thriving at work^5^ showed that supportive structural conditions—such as positive coworker relations, supportive leadership and organisational support—were positively associated with thriving, which in turn related to better health outcomes, including lower burnout and higher job satisfaction. Subsequent reviews confirmed that a supportive climate and empowering leadership are key antecedents of thriving.^24 25^ Empirical studies in nursing have similarly found that greater access to structural empowerment, such as adequate resources, organisational support and authentic leadership, is linked to higher levels of thriving.^711 26^,^30^ Moreover, thriving has been identified as an indirect relationship between work environment factors and outcomes such as well-being, performance and turnover intentions.^29 31 32^ However, few studies have examined stress symptoms as a specific outcome, and only one has suggested and examined thriving as an indirect relationship^11^ between structural empowerment and stress symptoms, revealing a critical gap in current knowledge.

The present study advances theoretical understanding by integrating structural empowerment with thriving at work into a unified model that explains how supportive work environments may protect nurses’ psychological well-being. Based on the Socially Embedded Model of Thriving at work and earlier research, we formulated the following hypotheses.

Hypothesis 5: Higher structural empowerment is related to higher thriving at work among RNs.

Hypothesis 6: Higher thriving at work is related to fewer stress symptoms among RNs.

Hypothesis 7: There is an indirect relationship between structural empowerment and perceived stress symptoms through thriving at work (figure 1).

In summary, structural empowerment, professional competence and thriving at work have all been found to be related to perceived stress symptoms. Furthermore, the structural empowerment theory and the socially embedded model of thriving at work, as well as earlier research, support the indirect relationships proposed in our study. Thus, the present study aimed to examine the relationship between structural empowerment, professional competence, thriving at work and perceived stress symptoms among registered Chinese nurses. Furthermore, professional competence and thriving were examined as indirect relationships between structural empowerment and stress symptoms.

## Methods

### Design

A cross-sectional correlational study was conducted. Although thriving at work and nurse professional competence are conceptually related, the Socially Embedded Model of Thriving at Work does not specify the directional relationship between them. Furthermore, previous empirical studies have not provided evidence that the proposed indirect relationships causally influence one another. Therefore, a parallel multiple mediation model was employed to examine the direct and indirect relationships of structural empowerment on stress symptoms. The study is part of a research project on nurses’ quality of work life, where a previous publication focused on person-centred care.^33^

### Participants

A convenience sample of 2568 eligible RNs working in three hospitals was invited to participate. In total, 2172 nurses from 121 departments responded to the questionnaire during the survey period (April and October 2023), with a response rate of 83.9%. The exclusion criteria were RNs on maternity, sick leave or on leave for further education during data collection.

### Data collection

Data were collected after obtaining permission from the nursing department deans of the participating hospitals. Unit lists and contact information for head nurses were provided by the deans. The first author then contacted each head nurse to arrange data collection. The head nurses were responsible for distributing the paper-based questionnaires to all RNs in their units and facilitating their collection. After completing the questionnaires, nurses personally deposited them into a locked collection box that allowed insertion only. The key to the box was held by the first author to ensure confidentiality. Two reminders were issued to increase the response rate. In total, 2172 valid questionnaires were returned, yielding a response rate of 83.9%.

### Measurement

Instruments used were the Conditions of Work Effectiveness Questionnaire^4^ Chinese version^34^ to measure structural empowerment, Nurse Professional Competence Scale short form^35^ Chinese version^36^, the Thriving at Work Scale^37^ Chinese version^38^ and one factor from the Psychosomatic health aspects questionnaire to measure perceived stress symptoms.^36^ The Conditions of Work Effectiveness Questionnaire and the Thriving at Work Scale were used with permission from the original developers. The Nurse Professional Competence Scale (short form) and the Psychosomatic Health Aspects Questionnaire were developed by people in our research team together with others; researchers interested in using these instruments may contact the corresponding author for permission and for use of the Nurse Professional Competence Scale see its home page.^39^ All instruments are validated with good psychometric properties (see table 1 for details).

**Table 1:** Details of instruments used in the study

Instruments and factors (Cronbach’s α values in the present study)	Description and examples of items	Calculation for the total and factor scores
Conditions of Work Effectiveness Questionnaire II^3 46^	Structural empowerment means structures within organisations important to the growth of empowerment.^5^	Item response alternatives range from 1 (never) to 5 (very often). The mean score is calculated for each factor, and the factors are summed for total score of the scale. Higher scores indicate a higher level of empowerment.
- Opportunities (three items, α=0.93)	Having access to possibility for growth within the organisation and opportunity to improve knowledge and skills. For instance, tasks using all your skills and knowledge.	
- Information (three items, α=0.97)	Having access to formal and informal knowledge necessary for the job. For instance, the goals of top management.	
- Support (three items, α=0.98)	Having access to support such as receiving feedback and guidance from other staff members. For instance, helpful hints or problem-solving advice.	
- Resources (three items, α=0.94)	Having access to materials and time required to do the job. For instance, time available to do necessary paperwork.	
- Formal power (three items, α=0.90)	Job characteristics such as: flexibility, visibility and centrality of the work. Such as, the amount of flexibility in my job.	
- Informal power (three items, α=0.90)	Networks (with other staff members) within and/or outside the organisation that influence empowerment. For instance, collaborating on patient care with physicians.	
Nurse Professional Competence Scale short form^36 38^	The scale is based on formal competence requirements for nurses developed by the Swedish National Board of Health and Welfare (2005) and also on core competences for nurses (WHO, 2009).	Item response alternatives range from 1 (very low) to 7 (very high). The total and factor scores are summarised as raw scores, divided by the highest possible total or factor score, and multiplied by 100. Higher scores indicate a higher level of competence.
- Nursing care (five items, α=0.97)	Items such as catering for the patient’s needs regarding basic, physical nursing care.	
- Value-based nursing care (five items, α=0.97)	Items such as showing concern and respect for the patient’s autonomy, integrity and dignity.	
- Medical and technical care (six items, α=0.97)	Items such as independently administer prescriptions.	
- Care pedagogics (five items, α=0.97)	Items such as inform and educate patients and next of kin individually.	
- Development and administration of nursing care (eight items, α=0.98)	Items such as, comply with existing regulations as well as guidelines and procedures.	
- Development, leadership, and organisation of nursing care (six items, α=0.98)	Items such as, act adequately in case of unprofessional conduct by staff.	
Thriving at Work Scale^35 53^	Thriving at work means a sense of having energy available and greater understanding and knowledge.^6^	Item response alternatives range from 1 (strongly disagree) to 7 (strongly agree). The mean score is calculated for each factor and total scale. Higher scores indicate a higher level of thriving.
- Vitality (five items, α=0.82)	Experiencing growth including both a sense of energised and alive. For instance, at work, I have energy and spirit.	
- Learning (five items, α=0.85)	A sense of continually improving. For instance, at work, I find myself learning often.	
Psychosomatic health aspects questionnaire ^[^^36^^]^ (10 items, α=0.92)	For instance, headache/stomach disorders caused by work; feeling unhappy and depressed.	Item response alternatives range from 0 (never) to 4 (very often). Total scores of stress symptoms are sums of the items, divided by the highest possible score of perceived stress and multiplied by 100. Higher scores represent a higher stress level.

### Confounding variables

The demographic characteristics related to mental health among hospital nurses in previous literature were considered as potential confounding variables. These included age,^40^,^43^ sex,^41^,^43^ marital status,^40 41 43^ educational level,^43^ years of clinical experience and^41 42^ night shift.^41 42^

### Data analysis

A total of 78 responses were excluded because of missing values for 50% or more items within the structural empowerment, thriving, nurse professional competence and stress symptom factors. For other missing values, less than 50% of items within each factor—structural empowerment (8 responses), thriving (54 responses), nurse professional competence (18 responses) and stress symptoms (84 responses)—were replaced by the series mean values of each item. The overall rate of missing data across all items was less than 1%. Given the very low level of missingness, mean imputation was used to preserve sample size and analytical consistency. In total, 1979 responses were included in the adjusted hypothesis analysis.

Data were analysed using IBM SPSS Statistics (V.28.0) and PROCESS (V.4.0) software. A student’s t-test, one-way analysis of variance and Pearson’s correlation analysis were conducted to test the association between participant characteristics and stress symptoms. Variables with p values less than 0.1 (marital status, educational level, clinical experience and night shift) were included as control variables in the mediation model, supplemented by sex based on theoretical considerations. Although age is conceptually relevant, it was excluded from the mediation model because of its high collinearity with clinical experience (see table 2), which could otherwise distort the parameter estimates. The PROCESS Model 4 with a parallel mediation model was employed to test the direct and indirect relationships of structural empowerment on stress symptoms. The indirect relationships were estimated using 5000 bootstrapping tests with 95% CIs, with zero falling outside the 95% CIs indicating statistical significance. Bootstrapping with 5000 resamples, as recommended by Hayes,^44^ was applied to estimate the indirect relationships, providing more accurate inferences and greater statistical power.

**Table 2:** Pearson correlation matrix of the study variables (N=2172)

Variables	1	2	3	4	5	6
1. Age (years)	--					
2. Clinical experience (years)	0.96^***^	--				
3. Structural empowerment	0.10^***^	0.09^***^	--			
4. Nurse professional competence	0.15^***^	0.15^***^	0.54^***^	--		
5. Thriving at work	0.05^*^	0.06^**^	0.58^***^	0.48^***^	--	
6. Stress symptoms	−0.18^***^	−0.18^***^	−0.43^***^	−0.34^***^	−0.45^***^	--

***p<0.001, **p<0.01, *p<0.05. Age was excluded from the mediation model due to its high collinearity with clinical experience.

## Results

### Demographic characteristics of participants

Table 2 shows participant characteristics; the majority were female (95.9%) with mean age being 30.3 (SD=7.7) years. Age (r=−0.18, p<0.001) and clinical experience (r=−0.18, p<0.001) had a significant association with stress symptoms (table 2). A significant difference in the stress symptoms scores according to marital status (t=6.21, p<0.001) and work shift (F=29.89, p<0.001) was also noted.

**Table 3:** Participant characteristics (N=2172)

Variables		Mean (SD)/n (%)
Age (years)		30.3 (7.7)
Clinical experience (years)		9.5 (8.0)
Sex	Female	2069 (95.9%)
	Male	88 (4.1%)
Marital status	Single/divorced/widow(er)	1066 (49.8%)
	Married	1075 (50.2%)
Educational level	Diploma	47 (2.2%)
	Associate degree	620 (29.1%)
	Bachelor or higher degree	1467 (68.7%)
Night shift	None	638 (30.3%)
	1–2 times /week	938 (44.6%)
	3 times or more /week	528 (25.1%)

Note: When sum does not add up to N=2172, there are internal missing data.

### Relationships between structural empowerment, professional competence, thriving and stress symptoms

**Figure 2:**
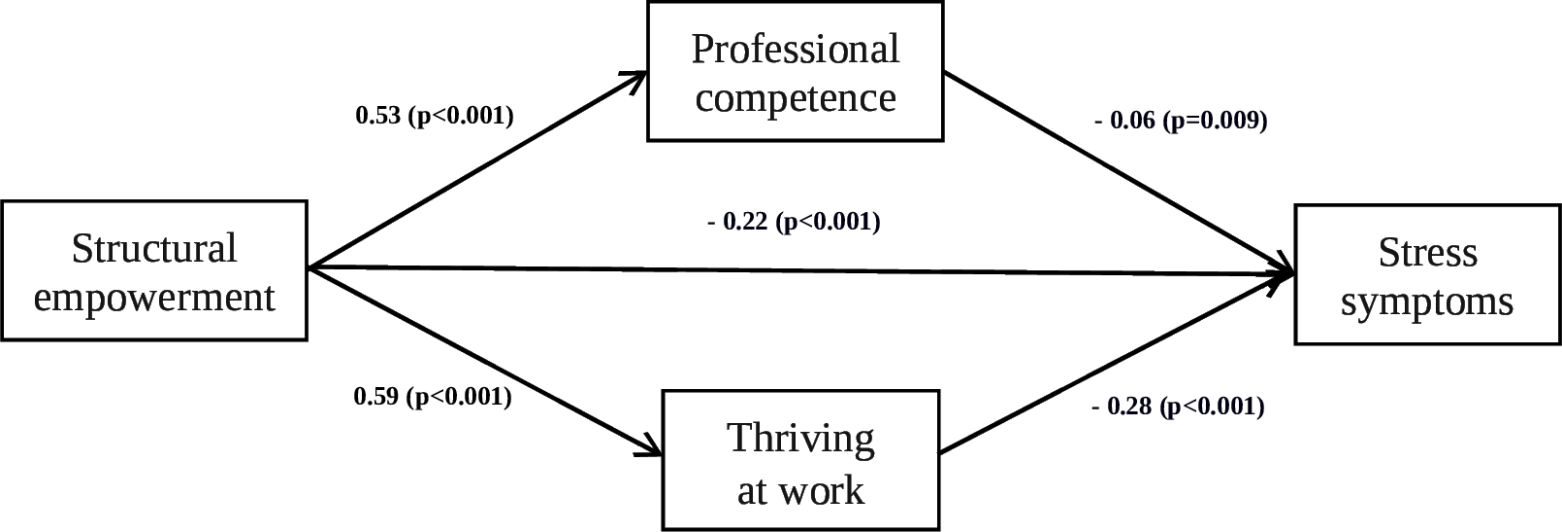
Final model, standardised coefficients. Note: Controlling marital status, clinical experience and work shift

Table 4 and figure 2 present the associations among the study variables. The regression results showed that structural empowerment was negatively related to stress symptoms (β=−0.42, p<0.001), and positively related to professional competence (β=0.53, p<0.001) and thriving at work (β=0.59, p<0.001), supporting H1, H2 and H5, respectively. Moreover, professional competence (β=−0.06, p=0.009) and thriving at work (β=−0.28, p<0.001) were negatively related to stress symptoms, supporting H3 and H6, respectively.

**Table 4:** Total, direct and indirect relationships of structural empowerment on stress symptoms (N=1979)

Model paths	Standardised estimated effect	P value	95% CI
Standardised total effect (H1)	−0.42	<0.001	
Standardised direct effect	- 0.23	<0.001	
Standardised indirect effect	−0.198		−0.235 to 0.162
Via professional competence (H4)	−0.033		−0.059 to to 0.009
Via thriving at work (H7)	−0.164		−0.204 to to 0.126
Controlling variables			
Sex	0.034	0.091	
Marital status	−0.034	0.199	
Educational level	0.080	<0.001	
Clinical experience	−0.130	<0.001	
Work shift	0.082	<0.001	

CI, confidence intervals; H, hypothesis.

### Parallel multiple mediation analysis

The results revealed that the parallel multiple mediator model accounted for 22.7% of the variation in stress symptoms (confounding variables were sex, marital status, educational level, clinical experience and night shifts). The total effect/relationship of structural empowerment on stress symptoms was −0.42 (p<0.001), and the direct effect/direct relationship was −0.23 (p<0.001). The total indirect effect/indirect relationship of structural empowerment on stress symptoms was −0.198 (95% CIs −0.235 to –0.162); through professional competence was −0.033 (95% CIs −0.059 to –0.009); and through thriving at work was −0.164 (95% CIs −0.204 to –0.126) (table 4).

## Discussion

This study contributes to Kanter’s structural empowerment theory and Spreitzer’s socially embedded model of thriving at work. Professional competence and thriving at work played parallel indirect relationships between structural empowerment and stress symptoms. The findings support the proposed hypotheses and provide insights into how to manage high levels of work-related stress symptoms to improve staff well-being.

As expected, our results were consistent with previous studies that showed that better access to structural empowerment was related to fewer stress symptoms^8 12 17^ (H1), higher competence^7 18^ (H2) and thriving^7 24 26 29^ (H5). These findings are in line with Kanter’s structural empowerment theory, which states that a work environment with good access to information, support, resources and opportunities is important for employees’ well-being.^5^ Further, the findings are also in line with Spreitzer’s model of thriving at work that contextual factors at work (cf., structural empowerment) improve staff thriving at work, which in turn improves staff health.

Our results suggest that a high level of structural empowerment is associated with a higher level of professional competence and thus associated with fewer stress symptoms (higher scores on the stress factor were related to fewer stress symptoms). These findings are consistent with previous research that found positive associations between structural empowerment and professional competence,^7 18^ and between professional competence and staff well-being.^21 22^ This study is one of the first to explore the indirect relationship of professional competence on the relationship between nurse-rated structural empowerment and stress symptoms. An earlier study of university professors also found that the relationship between working conditions and stress symptoms was mediated by perceived competence.^45^ This may be because when staff work in an empowering structure with good access to support, such as feedback and information on how the work should be performed, good access to resources and opportunities for development and growth,^5^ their professional competence improves,^7^ which in turn gives better self-confidence and fewer work-related stress symptoms.^46^ Research has shown links between professions with longer education and more stress. For example, physicians rate more stress than nurses,^47 48^ whereas increased competence within one’s profession has been shown to be related to fewer stress symptoms.^21^ Individuals with an expanded capability to perform tasks are linked to positive well-being.^22^ Professional competence may protect an individual’s physical and mental health.^21^ Thus, to alleviate the stress symptoms of nurses and other healthcare professionals, managers need to consider not only structural empowerment but also improved professional competence. In the present study, a higher level of education was associated with greater stress, consistent with previous findings showing that nurses with a higher educational level report greater work-related stress^47^ and that physicians tend to experience higher stress levels than nurses.^48^ These results demonstrated that, both between and within professions, longer education is generally linked to higher stress levels, which might be related to higher external and internal expectations for those with nurse specialist education or higher academic degree within nursing.

In addition, the results of our study indicate that the relationship between structural empowerment and stress symptoms had an indirect relationship through thriving at work. These findings are in line with previous studies showing that structural empowerment is linked with thriving at work,^7 24 26 49^ which in turn is related to staff well-being.^10 29^ Moreover, our results can be explained by using the socially embedded model of thriving at work by Spreitzer *et al.* According to this model, individuals who work within resource-rich social structures are energised and invest more effort in work, resulting in a higher level of thriving at work, which in turn might be linked to self-development and health.^6^ Thriving at work is a situational mechanism that links positive effects of social structures with better health outcomes.^6^ When individuals are embedded in a social structure with good access to information, support, resources and opportunity, they feel energised and eager to learn with a higher level of thriving at work.^6 7 24^ Meanwhile, when individuals are active at work, they produce more resources in their work, which further stimulates their thriving.^6^ Additionally, thriving at work, which is a pleasurable psychological state,^6^ plays a transmitting role in enhancing well-being and alleviating mental health problems.^10 29^ The model emphasises that both a sense of vitality and learning are essential for thriving, as thriving suffers when staff learn without vitality.^6^ One study also found that increased learning increased nurses’ risk of leaving their profession when vitality and resources were held constant,^50^ that is, thriving suffers when nurses learn without increased vitality.

A key finding of this study is that the indirect relationship of structural empowerment on stress symptoms through thriving at work (effect size=−0.164) was substantially stronger than through professional competence (effect size=−0.033). This indicates that while both indirect relationships contribute to stress reduction, thriving at work plays a more prominent role. Thriving encompasses affective and cognitive dimensions—such as engagement, energy and personal growth—which may more effectively relate to nurses’ stress,^6^ whereas professional competence primarily reflects knowledge and skills,^35^ which has a weaker relationship. Moreover, in the present study, professional competence and thriving were moderately related (r=0.48, p<0.001), suggesting interrelated rather than independent pathways. Structural empowerment may enhance both competence and thriving, with thriving potentially serving as the mechanism through which competence alleviates stress. According to the Socially Embedded Model of Thriving at Work, knowledge gained through work can precede thriving, which in turn fosters learning and competence.^6^ However, the theory does not specify a causal direction between thriving and professional competence. Future research should use structural equation modelling (SEM) to examine the interplay between these mediators in mitigating nurses’ stress.

### Implication for practice

Our study provides evidence for effective strategies for alleviating stress symptoms among nurses. Strategies that can promote nurses’ structural empowerment to decrease their stress symptoms include recognition of their central and important roles, promoting effective communication channels, good access to necessary resources and supplies for work, facilitating training and personal growth, and promoting interdisciplinary networks for staff within and outside the organisation.^51^ Since professional competence and thriving at work are significant links of the structural empowerment and stress symptom relationship, a wide range of interventions should be adopted to improve professional competence and especially thriving at work among nurses. Designing and implementing strategies to cultivate nurses’ professional competencies, such as education programmes with narratives and other reflective strategies by nursing managers, is necessary.^52^ The findings also suggest that it is vital for nursing managers to integrate thriving at work into the human resource management process to guide nurses in understanding the value of active learning and a sense of validity. Moreover, an integrated intervention programme should be considered to alleviate nurses’ stress symptoms, including providing access to empowering conditions, culturing professional competence and training in thriving at work.

### Limitations

This study has several potential limitations. First, its cross-sectional design limits the ability to draw causal inferences; longitudinal data are needed to further examine causal relationships. Second, all variables were measured through self-report instruments, which may introduce self-report bias. Third, although the PROCESS macro was appropriate for the current mediation analysis, future research could employ a latent SEM approach to account for measurement error and assess model fit. Moreover, nurses within the same departments or hospitals may share similar leadership, staffing, culture and resources, which could result in correlated responses. Ignoring such clustering may lead to underestimated standard errors and inflated type I error in mediation analysis. Future research could apply multilevel mediation models or latent multilevel SEM approaches to account for clustering effects and improve the robustness of the findings. Additionally, the mediation model explained a moderate proportion of variance in stress symptoms (R²=22.7%), and unmeasured factors may have contributed to the unexplained variance. Future research should include additional organisational, psychological or contextual variables to gain a more comprehensive understanding of stress among nurses. Fourth, the results could be biased when group mean was used to substitute missing data even if the amount of missing data was small. Finally, as participants were recruited from three hospitals in a single region of China, the findings may not be generalisable to other settings.

## Conclusions

This study investigated the relationship between structural empowerment and stress symptoms in hospital nurses. After adjusting for covariates, structural empowerment remained associated with stress symptoms, and nurses’ professional competence and thriving at work showed indirect relationships. Professional competence and thriving at work may represent possible pathways linking structural empowerment with stress symptoms. Nurse managers can alleviate nurses’ stress symptoms by providing good access to empowering structures, education programmes that cultivate professional competence and training for thriving at work.

## Data Availability

The data that support the findings of this study are available on request from the corresponding author. Due to confidentiality considerations, individual-level data cannot be publicly shared. However, metadata and aggregated subsets of the data, which do not contain personal identifiers, can be made available to interested researchers.
