# Supplementary material for: Direct and indirect relationships between structural empowerment, professional competence, thriving at work and perceived stress symptoms: a cross-sectional correlational study on hospital nurses in a Chinese province
Source: BMJ Open. 2025 Dec 25;15(12):e100696. doi: 10.1136/bmjopen-2025-100696 (PMC12742184; doi:10.1136/bmjopen-2025-100696)
Supplement: Supplementary file 1 [file bmjopen-2025-100696.reviewer_comments.pdf]

PEER REVIEW HISTORY

BMJ Open publishes all reviews undertaken for accepted manuscripts. Reviewers are asked to complete a checklist review form and are provided with free text boxes to elaborate on their assessment. These free text comments are reproduced below.

ARTICLE DETAILS

Title (Provisional)

The direct and indirect relationships between structural empowerment, professional competence, thriving at work and perceived stress symptoms– A cross-sectional correlational study on hospital nurses in a Chinese province

Authors

Xu, Lijuan; Nilsson, Annika; Zhu, Kewen; Engström, Maria

VERSION 1 - REVIEW

**Reviewer**	**1**
**Name**	**Dewi, Ayu Putu Madri**
**Affiliation**	**Amsterdam UMC Location AMC, Epidemiology and Data Science**
**Date**	**17-Mar-2025**
**COI**	**None**

I am an Open Science researcher, who has checked your manuscript for its adherence to a number of Open Science practices.

Below you find the checklist that I used to assess your manuscript for adherence to a number of these practices.

Based on this assessment, I would suggest the following refinements/additions:

1. *Availability of a protocol: Please provide a (link to the) the study's protocol, if available.*
2. *Pre-registration: Please provide information on whether and where the study was pre-registered.*
3. *Open Materials: Please provide whether and how the materials (such as questionnaires) used in your study can be shared.*
4. *Open Data: the authors state "The data that support the findings of this study are available on request from the corresponding author." Given the potential confidentiality of the data, please specify whether de-identified, metadata, or a subset of the data can still be made publicly available.*
5. *Open Code: Please provide (a link to) the code used for the analyses in this study.*
6. *Citations: Please provide citations for your materials/data/codes.*

7. *Software and tools used are described: OK.*
8. *Open access publication: OK.*
9. *Reporting checklist provided: OK.*
10. *Preprint: Please state explicitly whether and where the preprint of this study can be found.*

**Reviewer**	**2**
**Name**	**Jaracz, Krystyna**
**Affiliation**	**Poznan University of Medical Sciences**
**Date**	**28-Jul-2025**
**COI**	**None**

Thank you the Editor for the opportunity to review this manuscript .

Introduction

Overall, the study addresses the important issue of how organizational structures, the social environment, and personal resources of hospital nurses influence their psychological well-being.

The theoretical framework is clearly and effectively presented in the introduction, particularly the psychological constructs of Kanter's Structural Empowerment Theory and Spreitzer's Socially Embedded Model of Thriving at Work, which are relatively less known in the nursing literature. The authors also provide a solid conceptual foundation for the hypotheses, focusing on the relationships between structural empowerment and stress symptoms, with thriving at work and professional competence as mediators, and controlling for potential confounders.

The research assumptions are relevant; however, the hypotheses appear somewhat self-evident. It is generally expected that more supportive working conditions and higher professional competence would be associated with improved psychological well-being among nursing staff, rather than the reverse.

In my opinion, the rationale for testing the proposed theoretical model is not fully convincing. It would be beneficial to explicitly state whether the authors aim to explore previously unexamined relationships, expand or integrate existing theories, or test counterintuitive assumptions. Furthermore, it remains unclear what novel insights this model offers to the understanding of the nursing work environment and to the broader body of research on this topic.

Some of these aspects are briefly mentioned in the discussion; however, key arguments from a scientific standpoint appear dispersed and lack depth. I would recommend consolidating this content into a coherent paragraph in the introduction, clearly presenting a critical

rationale that identifies the knowledge gap in the nursing literature and outlines the specific contributions of the study. This would significantly enhance the scientific merit and relevance of the manuscript.

Participants

I suggest providing more detailed information about participant recruitment and the method of survey administration (e.g., paper-based or online with the medium used). In short, it would be useful to clarify how the study was organized and how the questionnaires were distributed.

Data Analysis

It would be helpful to include a brief explanation of why bootstrapping was applied and what type of bootstrapping was used.

Results

The findings are presented clearly and understandably. I have no questions or concerns regarding this section.

Discussion

If the authors decide to revise the introduction, the discussion section should be adjusted accordingly to ensure coherence with the revised conceptual framing.

To sum up: The manuscript is well-written and carefully prepared. I have no concerns regarding the presentation or interpretation of the results. However, I encourage the authors to adopt a more critical and analytical approach in the introduction - representing the conceptual phase of the research process - in order to better balance the confirmation of hypotheses with the advancement of their theoretical understanding.

**Reviewer**	**3**
**Name**	**Brown, Roger**
**Affiliation**	**University of Wisconsin Madison, Research Division**
**Date**	**08-Sep-2025**
**COI**	**None**

---

1. Your table 2 should be table 1 description of your sample, which is typical format.

2. Not knowing the extent of within scale missingness, mean imputation may bias estimates and reduce variability. More robust methods (e.g., multiple imputation or full-information maximum likelihood) would be preferable.

3. Confounder selection based on bivariate significance is data-driven and may omit conceptually relevant factors (e.g., age, education, sex). This approach risks residual confounding.

4. Explained variance (R^2 = 22.2%) is moderate; unmodeled variables may account for much of the variance in stress symptoms.

5. Scaling interpretation (e.g., "higher scores = fewer stress symptoms") is counter intuitive and requires care, as readers may struggle with this.

6. Table 4, confused about your 95% Cis, are these for unstandardized estimates, should be consistent with the reported estimates in the table. Also, I think this table could be cleared up a little for the reader. Indication of the total effect, the total indirect effect, and specific indirect effects, it is all there but just a little unclear.

7. Minor point, convention is that confidence intervals be separated by a comma, not a dash, so as to reduce confusion with negative values.

8. Nurses in the same department most likely share leaders, staffing, culture, and resources. These very constructs are tied to empowerment and thriving, so responses will be correlated (ICC > 0) to some degree. Ignoring this typically underestimates SEs and inflates Type I error for paths in the mediation model. The authors might consider fitting a 2-level multilevel mediation (department → nurse) model, and at the very least, apply cluster-robust SEs and report ICCs or design effects to show that clustering has been addressed. Or consider this a limitation.

9. Parallel mediation assumes that the mediators are independent conditional on the predictor. In your study, professional competence and thriving at work are conceptually and empirically correlated. Table 3 shows that competence and thriving are correlated at $r = 0.48$ ($p < 0.001$), which seems to indicate they are not independent pathways. Treating them as independent pathways may overstate the uniqueness of each indirect effect. If they are causally related (e.g., competence → thriving), then a sequential (serial) mediation model may be more appropriate. The use of parallel mediation is appropriate, and PROCESS does take mediator correlation into account by estimating each mediator's unique contribution. However, when mediators are moderately correlated, as appears to be the case here, their unique indirect effects can become less stable, with larger standard errors or suppression effects. This does not invalidate the analysis, but it does mean the indirect effects should be interpreted cautiously. A latent structural equation modeling (SEM) framework would allow the correlation between mediators to be modeled directly and would provide a clearer picture of whether the mechanisms are best understood as parallel, serial, or overlapping.

10. While the use of the PROCESS macro provides a straightforward estimation of indirect effects, the large sample size and the availability of multi-item validated instruments strongly justify the use of a latent structural equation modeling (SEM) approach. SEM would have permitted correction for measurement error, assessment of global model fit, and explicit testing of competing mediation structures, providing more robust evidence for the hypothesized pathways.

General comment: The analytic approach using PROCESS macro is appropriate and produces interpretable estimates of mediation. However, given the large sample size and the use of multi-item validated instruments, the study could have benefited from a latent structural equation modeling (SEM) framework. SEM would allow correction for measurement error, provide global model fit indices, and formally compare alternative mediation structures (e.g., parallel vs. serial pathways between professional competence and thriving). In addition, the sample was clustered within 121 departments, and the use of single-level mediation models may underestimate standard errors; a multilevel approach or at minimum cluster-robust corrections would more fully account for this design feature. These limitations do not undermine the overall findings, but it would strengthen the manuscript if the authors explicitly acknowledged them in the limitations section and noted that future work may employ latent multilevel SEM approaches to optimize rigor.

**Reviewer**	**4**
**Name**	**He, Liping**
**Affiliation**	**Universiti Putra Malaysia**
**Date**	**30-Sep-2025**
**COI**	**None**

I thank the authors for this interesting manuscript on structural empowerment and perceived stress symptoms of hospital nurses. The study is well-motivated, and the dataset is valuable. However, several issues need to be addressed before publication, primarily concerning the methodology and the interpretation of the results.

1. In the Method, confounding variables included age, sex, marital status, educational level, years of clinical experience, and night shifts. However, the educational level is missing in Table 2.If the educational level was omitted because it was not statistically significant, this should be clearly stated in the Results section.

2. In the third paragraph of the discussion, you cited that professions with longer education (e.g., physicians vs. nurses) are linked to higher stress [45,46]. However, the authors' own results indicated that the individual education level within the nursing sample was not a significant factor. The argument presents a potential logical inconsistency that requires clarification.

3. The results present a compelling finding that merits deeper discussion: the indirect effect through thriving at work (effect size = 0.166) is substantially stronger than the effect through professional competence (effect size = 0.030). However, the discussion section treats these two pathways in parallel without comparing their relative importance or exploring the potential reasons for this notable difference. To strengthen the manuscript, it is essential to elaborate on this disparity in the Discussion section.

4.The introduction and background sections of the manuscript are comprehensive. However, their overall length is somewhat excessive, and the connection of some content to the core research question could be strengthened. It is recommended that the author streamline and focus these sections, more directly guiding the discussion toward the research gap and the aims of this study, thereby enhancing the readability and impact of the article.

5. In the data analysis section, the manuscript states: "Results with p-values less than 0.1 (marital status, clinical experience, and night shift) were then used as control variables in the mediation model." Please confirm whether educational level was considered as a potential confounding variable in this initial screening step, given that it was not included in the final model.

VERSION 1 - AUTHOR RESPONSE

Responses to Reviewer 1

（Open Science researcher, who has checked your manuscript for its adherence to a number of Open Science practices） Ms. Ayu Putu Madri Dewi, Amsterdam UMC Location AMC

I have some feedback related to open science practices.

Below you find the checklist that I used to assess your manuscript for adherence to a number of these practices. Based on this assessment, I would suggest the following refinements/additions:

Thank you very much. Useful to reflect on and reminds us of pre-registration also for observational studies, which we had not done for this project.

1. Availability of a protocol: Please provide a (link to the) the study's protocol, if available.

Response: This study did not have a preregistered or published protocol.

2. Pre-registration: Please provide information on whether and where the study was pre registered.

Response: This study was not pre-registered. However, it is something we need to remember for future research projects.

3. Open Materials: Please provide whether and how the materials (such as questionnaires) used in your study can be shared.

Response: Thank you. Regarding the materials used in our study:

① Conditions of Work Effectiveness Questionnaire and Thriving at Work Scale were used with permission from the original developers.

② Nurse Professional Competence Scale (short form) and the psychosomatic health aspects questionnaire measuring perceived stress symptoms were developed by persons in our

research team together with others. Researchers interested in using these instruments can contact us directly to obtain permission for use. For the Nurse Professional Competence Scale there is also a homepage that describes what to do if interested to use the instrument.
We have updated the manuscript in the **Methods section under "Measurement"** to clarify the availability and authorization of all questionnaires.

4. Open Data: the authors state "The data that support the findings of this study are available on request from the corresponding author." Given the potential confidentiality of the data, please specify whether de-identified, metadata, or a subset of the data can still be made publicly available.

Response: In our study, the dataset contains sensitive information from participated nurses, including personal records, which cannot be made fully public due to privacy concerns. To address the reviewer's comment, we have clarified the availability of data updated in the **Declaration section** at the end of the manuscript.

5. Open Code: Please provide (a link to) the code used for the analyses in this study.

Response: Thank you. In this study, all statistical analyses were performed using IBM SPSS Statistics and PROCESS macro. These analyses do not involve Code; rather, we used standard procedures within these software packages. Therefore, no code is available for sharing.

6. Citations: Please provide citations for your materials/data/codes.

Response: Thank you very much for your comment regarding citations.

① Materials/Questionnaires: The questionnaires used in this study were previously validated instruments, which are already cited in the original manuscript.

② Data: Since this study was submitted we have got a paper published from the same research project/data collection, this study is now cited in the manuscript. We utilized secondary data in this study, which have now been appropriately referenced with relevant citations in **Method section "Design"**.

③ Code: Our study did not involve any computational code; therefore, no citation is applicable for code.

7. Software and tools used are described: OK.

Response: Thank you.

7. Open access publication: OK.

Response: Thank you.

8. Reporting checklist provided: OK.

Response: Thank you

10. Preprint: Please state explicitly whether and where the preprint of this study can be found

Response: We would like to clarify that this study has not been posted as a preprint. Therefore, no preprint version is available. We have added a note in the **Declaration**.

Response to Reviewer 2

Dr. Krystyna Jaracz, Poznan University of Medical Sciences

Comments to the Author:

Introduction

Overall, the study addresses the important issue of how organizational structures, the social environment, and personal resources of hospital nurses influence their psychological well-being.

The theoretical framework is clearly and effectively presented in the introduction, particularly the psychological constructs of Kanter's Structural Empowerment Theory and Spreitzer's Socially Embedded Model of Thriving at Work, which are relatively less known in the nursing literature. The authors also provide a solid conceptual foundation for the hypotheses, focusing on the relationships between structural empowerment and stress symptoms, with thriving at work and professional competence as mediators, and controlling for potential confounders.

Response: We deeply appreciate your thoughtful and encouraging feedback. Thank you for acknowledging the significance of our research topic and for your positive assessment of the theoretical framework.

The research assumptions are relevant; however, the hypotheses appear somewhat self-evident. It is generally expected that more supportive working conditions and higher professional competence would be associated with improved psychological well-being among nursing staff, rather than the reverse.

In my opinion, t**he rationale for testing the proposed theoretical model is not fully convincing**. It would be beneficial to explicitly state whether the authors aim to explore previously unexamined relationships, expand or integrate existing theories, or test counterintuitive assumptions.

Response: Thank you for the insightful comments. We agree that the rationale for testing the proposed theoretical model needs to be more explicitly articulated. In response, we have clarified the theoretical purpose. We have explicitly stated that this study aims to integrate

and expand two theoretical perspectives—Kanter's Structural Empowerment Theory and Spreitzer's Socially Embedded Model of Thriving at Work—to examine their combined explanatory power for nurses' psychological wellbeing. See revisions in the **Background section**.

Furthermore, it remains unclear what novel insights this model offers to the understanding of the nursing work environment and to the broader body of research on this topic. Some of these aspects are briefly mentioned in the discussion; however, key arguments from a scientific standpoint appear dispersed and lack depth. I would recommend consolidating this content into a coherent paragraph in the introduction, clearly presenting a critical rationale that identifies the knowledge gap in the nursing literature and outlines the specific contributions of the study. This would significantly enhance the scientific merit and relevance of the manuscript.

Response: Thank you for your suggestion to clarify the novelty and scientific contribution of our proposed theoretical model. In response, we have revised the introduction to more clearly articulate the study's rationale, theoretical contribution, and knowledge gap within the nursing literature. See revisions in **Background section**.

Participants

I suggest providing more detailed information about participant recruitment and the method of survey administration (e.g., paper-based or online with the medium used). In short, it would be useful to clarify how the study was organized and how the questionnaires were distributed.

Response: Thank you very much. We apologize for the omission of details regarding data collection in the original manuscript. We have now added a description of the participant recruitment and survey administration process to the **Methods section (Data collection)**.

Data Analysis

It would be helpful to include a brief explanation of why bootstrapping was applied and what type of bootstrapping was used.

Response: Thank you for this valuable comment. Accordingly, we have added a short explanation in the **Data Analysis section** to specify the reason and type of bootstrapping applied.

Results

The findings are presented clearly and understandably. I have no questions or concerns regarding this section.

Response: Thank you very much for the positive feedback and acknowledging the clarity of our findings.

Discussion

If the authors decide to revise the introduction, the discussion section should be adjusted accordingly to ensure coherence with the revised conceptual framing.

To sum up: The manuscript is well-written and carefully prepared. I have no concerns regarding the presentation or interpretation of the results. However, I encourage the authors to adopt a more critical and analytical approach in the introduction - representing the conceptual phase of the research process - in order to better balance the confirmation of hypotheses with the advancement of their theoretical understanding.

Response: Thank you for the constructive feedback and for recognizing the quality of our manuscript. In response, we have revised the **Introduction** to adopt a more critical and analytical approach, emphasizing the conceptual rationale and theoretical underpinnings of the study, as well as the **Discussion section**.

Responses to Reviewer 3

Prof. Roger Brown, University of Wisconsin Madison

Comments to the Author:

1. Your table 2 should be table 1 description of your sample, which is typical format.

Response: Thank you. In our manuscript, Table 1 summarizes the study instruments described in the Methods section, while Table 2 presents participants' general characteristics in the Results section. Therefore, we respectfully retained the current table order to ensure consistency between the Methods and Results sections.

2. Not knowing the extent of within scale missingness, mean imputation may bias estimates and reduce variability. More robust methods (e.g., multiple imputation or full-information maximum likelihood) would be preferable.

Response: Thank you very much. In the revised manuscript, we have added information regarding the extent of missing data within each scale. As the proportion of missing values was very small (less than 1% for all items), we adopted mean imputation to maintain data completeness while minimizing potential bias. Given the low level of missingness, we considered mean imputation could be used. The relevant information has been added to the **Data Analysis section**. Text has also been added to the section, limitations. However, if you consider that we need to use multiple imputation we will use it.

3. Confounder selection based on bivariate significance is data-driven and may omit conceptually relevant factors (e.g., age, education, sex). This approach risks residual confounding.

Response: Thank you very much. We fully agree that confounder selection should consider both statistical and conceptual relevance. In response, we have clarified our rationale for variable selection in the revised manuscript. Specifically, we retained variables that were statistically significant at $p < 0.1$ (marital status, educational level, clinical experience, and night shift) and included sex based on theoretical relevance. However, age was not included in the mediation model due to its high collinearity with clinical experience (see Table 3). This clarification has been added to the **Methods section (Data analysis)**.

4. Explained variance ($R^2 = 22.2\%$) is moderate; unmodeled variables may account for much of the variance in stress symptoms.

Response: Thank you. We agree with you. This point has been added to the **Limitations section** of the revised manuscript, acknowledging that the explained variance ($R^2 = 22.7\%$) is moderate and suggesting that future studies should include additional variables to further explain stress symptoms.

5. Scaling interpretation (e.g., "higher scores = fewer stress symptoms") is counter intuitive and requires care, as readers may struggle with this.

Response: Thank you very much for your insightful suggestion. We fully agree that the scaling interpretation ("higher scores = fewer stress symptoms") could be potentially confusing for readers. As the stress scale used in this study was developed by people in our research team, we carefully discussed your suggestion and have adopted it. We have reversed the scoring direction of the stress scale so that higher scores now indicate a higher level of stress. The description of the scale has been revised in the manuscript to clearly state: "Higher scores represent a higher stress level." Accordingly, all stress-related hypotheses, results, including textual descriptions and tables, have been revised to reflect this change. See revisions in **Table 1,** and the **results** related to stress in the main text and **Table 4.**

6. Table 4, confused about your 95% Cis, are these for unstandardized estimates, should be consistent with the reported estimates in the table. Also, I think this table could be cleared up a little for the reader. Indication of the total effect, the total indirect effect, and specific indirect effects, it is all there but just a little unclear.

Response: Thank you very much. We have revised **Table 4** to ensure consistency and clarity. All results are now presented as standardized estimated effects, and the 95% CIs for

unstandardized estimates have been removed, as only the indirect effects included standardized 95% CIs in the statistical output. In addition, Table 4 has been slightly adjusted to improve readability and to more clearly present the total, total indirect, and specific indirect effects.

7. Minor point, convention is that confidence intervals be separated by a comma, not a dash, so as to reduce confusion with negative values.

Response: Thank you for the suggestion. We have revised all confidence intervals in the manuscript to use a comma as the separator instead of a dash. See revisions in **Table 4** and **the Result section** in Abstract and main text.

8. Nurses in the same department most likely share leaders, staffing, culture, and resources. These very constructs are tied to empowerment and thriving, so responses will be correlated (ICC > 0) to some degree. Ignoring this typically underestimates SEs and inflates Type I error for paths in the mediation model. The authors might consider fitting a 2-level multilevel mediation (department → nurse) model, and at the very least, apply cluster-robust SEs and report ICCs or design effects to show that clustering has been addressed. Or consider this a limitation.

Response: Thank you for this insightful comment. We agree that clustering effects within departments may influence the results, and this should be acknowledged. As a limitation, we have added a discussion of the potential for correlated responses among nurses in the same department in the **Limitation section.** We note that ignoring this clustering could lead to underestimated standard errors and inflated Type I error. Future studies could adopt a multilevel mediation model or apply cluster-robust standard errors to address this issue more rigorously.

9. Parallel mediation assumes that the mediators are independent conditional on the predictor. In your study, professional competence and thriving at work are conceptually and empirically correlated. Table 3 shows that competence and thriving are correlated at $r = 0.48$ ($p < 0.001$), which seems to indicate they are not independent pathways. Treating them as independent pathways may overstate the uniqueness of each indirect effect. If they are causally related (e.g., competence → thriving), then a sequential (serial) mediation model may be more appropriate.

Response: Thank you for the constructive comment. According to the Socially Embedded Model of Thriving at Work (used in the manuscript), the knowledge/competence produced in the doing of work is considered an antecedent of thriving at work, and thriving can in turn foster a sense of learning and enhanced competence. However, the theory does not explicitly

specify a directional or causal relationship between thriving at work and nurse professional competence. Moreover, previous studies have not provided empirical evidence supporting a causal influence of one mediator on the other. Therefore, in this study, we adopted a parallel multiple mediator model to examine the indirect effects of structural empowerment on stress, rather than a sequential mediation model.

We acknowledge that this rationale was not clearly described in the original manuscript. In the revised version, we have clarified this reasoning in the **Design section** to better justify the choice of the parallel mediation model. In addition, for the relationship between thriving and professional competence, we added a discussion in the revised manuscript in the **Discussion section**. Based on the comments, we ran an complementary analysis see bellow. In the analysis serial mediation analysis, the indirect relationship of structural empowerment on stress through thriving at work → professional competence was -0.009 (95% CI: -0.016, -0.002). While we would like to keep it as it is, as there is no theory to guide which one to put first in a sequential mediation analysis.

Supplementary file. Total, direct, and indirect relationships of structural empowerment on stress symptoms, with serial mediation analysis from thriving at work, professional competence.

**Model paths**	**Standardized estimated effect**	**P-value**	**95%CI**
Standardized total effect (H1)	- 0.42	<0.001	
Standardized direct effect	- 0.23	<0.001	
Standardized indirect effect	-0.198		- 0.234, - 0.160
Via thriving at work	-0.164		-0.202, - 0.127
Via professional competence	-0.024		-0.043, - 0.006
Via thriving at work -->professional competence	-0.009		-0.016, - 0.002
Controlling variables			
Sex	0.034	0.091	
Marital status	- 0.034	0.199	
Educational level	0.080	<0.001	
Clinical experience	- 0.130	<0.001	
Work shift	0.082	<0.001	

Note: [a] Bootstrap confidence intervals; CI confidence intervals, H hypothesis

The use of parallel mediation is appropriate, and PROCESS does take mediator correlation into account by estimating each mediator's unique contribution. However, when mediators are moderately correlated, as appears to be the case here, their unique indirect effects can become less stable, with larger standard errors or suppression effects. This does not invalidate the analysis, but it does mean the indirect effects should be interpreted cautiously. A latent structural equation modeling (SEM) framework would allow the correlation between mediators to be modeled directly and would provide a clearer picture of whether the mechanisms are best understood as parallel, serial, or overlapping.

Response: Thank you very much for your insightful suggestion. We agree with your comment and have clarified in the revised manuscript the rationale for using PROCESS in this study. PROCESS was chosen because it is less sensitive to data distribution assumptions compared to structural equation modeling (SEM). We acknowledge that SEM could provide a more comprehensive approach to modeling the correlations between mediators. However, we are not that familiar with running SEM. The limitation has been noted in the **Limitations section**, suggesting that future research could employ a latent SEM framework to further validate the findings.

9. While the use of the PROCESS macro provides a straightforward estimation of indirect effects, the large sample size and the availability of multi-item validated instruments strongly justify the use of a latent structural equation modeling (SEM) approach. SEM would have permitted correction for measurement error, assessment of global model fit, and explicit testing of competing mediation structures, providing more robust evidence for the hypothesized pathways.

General comment: The analytic approach using PROCESS macro is appropriate and produces interpretable estimates of mediation. However, given the large sample size and the use of multi-item validated instruments, the study could have benefited from a latent structural equation modeling (SEM) framework. SEM would allow correction for measurement error, provide global model fit indices, and formally compare alternative mediation structures (e.g., parallel vs. serial pathways between professional competence and thriving).

Response: We appreciate the reviewer' s insightful comment. We agree that employing a latent structural equation modeling (SEM) framework could offer additional advantages, such as accounting for measurement error, providing model fit indices, and allowing formal comparisons of alternative mediation pathways. We have acknowledged this as a limitation

and suggested that future studies consider adopting SEM to further validate and extend our findings. This revision has been incorporated into the **Limitations section** of the manuscript. As mentioned earlier we are not that familiar with running SEM.

In addition, the sample was clustered within 121 departments, and the use of single-level mediation models may underestimate standard errors; a multilevel approach or at minimum cluster-robust corrections would more fully account for this design feature. These limitations do not undermine the overall findings, but it would strengthen the manuscript if the authors explicitly acknowledged them in the limitations section and noted that future work may employ latent multilevel SEM approaches to optimize rigor.

Response: We appreciate the reviewer's thoughtful and constructive comment. We fully agree that the clustered data structure may lead to underestimated standard errors when using single-level mediation models. We have acknowledged this as a study limitation and noted that future research could apply multilevel mediation models or latent multilevel SEM approaches to more appropriately account for such clustering and enhance methodological rigor. This revision has been incorporated into the **Limitations section** of the manuscript.

Responses to Reviewer: 4

Dr. Liping He, Universiti Putra Malaysia

Comments to the Author:

I thank the authors for this interesting manuscript on structural empowerment and perceived stress symptoms of hospital nurses. The study is well-motivated, and the dataset is valuable. However, several issues need to be addressed before publication, primarily concerning the methodology and the interpretation of the results.

Response: Thank you very much for the thoughtful and encouraging feedback on our manuscript.

1. In the Method, confounding variables included age, sex, marital status, educational level, years of clinical experience, and night shifts. However, the educational level is missing in Table 2. If the educational level was omitted because it was not statistically significant, this should be clearly stated in the Results section.

Response: Thank you for your careful reading and valuable comment. The variable educational level was omitted from Table 2 in the previous version. We have now included educational level in both the descriptive results, **Table 2, and Table 4** to ensure consistency

with the confounding variables listed in the Methods section. The **Results section** has also been revised accordingly.

2. In the third paragraph of the discussion, you cited that professions with longer education (e.g., physicians vs. nurses) are linked to higher stress [45,46]. However, the authors' own results indicated that the individual education level within the nursing sample was not a significant factor. The argument presents a potential logical inconsistency that requires clarification.

Response: Thank you very much. We realized that the part describing the relationship between education and stress in our own results had been omitted. In our study, educational level was indeed found to be positively associated with stress (see **Table 4**), which aligns with the cited literature indicating that professions with longer education may experience greater stress. We have now revised the **Results and Discussion sections** accordingly to ensure logical consistency throughout the manuscript.

3. The results present a compelling finding that merits deeper discussion: the indirect effect through thriving at work (effect size = 0.166) is substantially stronger than the effect through professional competence (effect size = 0.030). However, the discussion section treats these two pathways in parallel without comparing their relative importance or exploring the potential reasons for this notable difference. To strengthen the manuscript, it is essential to elaborate on this disparity in the Discussion section.

Response: Thank you for this valuable comment. We fully agree with your observation. In the revised manuscript, we have expanded the **Discussion section** to (1) compare the two mediation effects, and (2) discuss the potential causal linkage between these two mediators. To address this point, we also added a statement suggesting that future studies should employ structural equation modeling (SEM) to further explore the interplay between the two mediators.

4.The introduction and background sections of the manuscript are comprehensive. However, their overall length is somewhat excessive, and the connection of some content to the core research question could be strengthened. It is recommended that the author **streamline and focus these section**s, more **directly guiding the discussion toward the research gap and the aims** of this study, thereby enhancing the readability and impact of the article.

Response: Thank you very much for the constructive feedback about the Introduction and Background sections. We agree that streamlining these sections would strengthen the connection to the core research question and improve readability. In response, we have carefully revised the manuscript to (1) condense background information by removing non-

essential details; and (2) clarify the linkage between existing literature and the research gap. See revisions in the **Background section**.

5. In the data analysis section, the manuscript states: "Results with p-values less than 0.1 (marital status, clinical experience, and night shift) were then used as control variables in the mediation model." Please confirm whether educational level was considered as a potential confounding variable in this initial screening step, given that it was not included in the final model.

Response: Thank you for your valuable comment. We confirm that educational level was indeed considered as a potential confounding variable in the initial screening step ($p = 0.10$). In the revised analysis, it has been included in the final mediation model. Moreover, revisions have been made to the **Data Analysis section**, **Results**, and **Table 4** to ensure full consistency in the description of the statistical methods and reported findings.

VERSION 2 - REVIEW

**Reviewer**	**2**
**Name**	**Jaracz, Krystyna**
**Affiliation**	**Poznan University of Medical Sciences**
**Date**	**30-Nov-2025**
**COI**	

I have no further comments or questions.

**Reviewer**	**3**
**Name**	**Brown, Roger**
**Affiliation**	**University of Wisconsin Madison, Research Division**
**Date**	**25-Nov-2025**
**COI**	

The authors have responded to my concerns.
